# *FTO* variant is not associated with osteoarthritis in the Chinese Han population: replication study for a genome-wide association study identified risk loci

**DOI:** 10.1186/s13018-018-0769-2

**Published:** 2018-04-02

**Authors:** Jin Dai, Pu Ying, Dongquan Shi, Huacheng Hou, Ye Sun, Zhihong Xu, Dongyang Chen, Guoqiang Zhang, Ming Ni, Huajian Teng, Yan Wang, Qing Jiang

**Affiliations:** 1 The department of Sports Medicine and Adult Reconstructive Surgery, Drum Tower Hospital, School of Medicine, Nanjing University, 321 Zhongshan Road, Nanjing, 210009 Jiangsu China; 2 Laboratory for Bone and Joint Diseases, Australia-China Joint Centre, Model Animal Research Center, Nanjing University, 12 Xuefu Road, Nanjing, 210061 China; 3 Department of Orthopaedics, Chinese PLA General Hospital, 28 Fuxing Road, Beijing, 100853 China

**Keywords:** *FTO*, Osteoarthritis, Obesity, Single nucleotide polymorphism, Body mass index

## Abstract

**Background:**

Osteoarthritis is the most prevalent form of arthritis worldwide and is the major cause of pain and loss of function in elderly people. A signal of the fat mass and obesity-associated (*FTO*) gene had been reported in a genome-wide association study of osteoarthritis. The *FTO* polymorphism (rs8044769) might exert its effect on osteoarthritis through obesity, because it was reported as a body mass index-associated single-nucleotide polymorphism. And replication studies showed inconsistent results for this association. Our present study is to check the association of rs8044769 with osteoarthritis and body mass index in Chinese Han population.

**Methods:**

A case-control association study was conducted by using 890 osteoarthritis cases and 844 controls in Chinese Han population. rs8044769 was genotyped in all subjects. Allelic and genotypic frequencies were compared between osteoarthritis cases and control subjects. Associations between rs8044769 and body mass index, and body mass index and osteoarthritis were also assessed.

**Results:**

No significant difference was detected in genotype or allele distribution between osteoarthritis cases and controls (*P* > 0.05). Stratification by gender and body mass index revealed negative association between rs8044769 and osteoarthritis. We did not find any solid association between rs8044769 and higher body mass index. Meanwhile, we demonstrated that higher body mass index (body mass index ≥ 25) was associated with osteoarthritis.

**Conclusion:**

Our present study suggested that rs8044769 was not associated with osteoarthritis susceptibility or higher body mass index, and higher body mass index was a risk factor for osteoarthritis in the Chinese Han population. We also proposed that stratification by clinical parameters was crucial to reduce false-positive result in OA association studies.

**Electronic supplementary material:**

The online version of this article (10.1186/s13018-018-0769-2) contains supplementary material, which is available to authorized users.

## Background

Osteoarthritis (OA; OMIM 165720) is the most common degenerative disease of the joints among the elderly. Hip and knee OA are the main causes of pain, functional loss, and depressed quality of life in both developed and developing countries. OA, like other complex chronic disease, develops through a combination of environmental and genetic risk factors [1].

Obesity has been consistently associated with an augmented risk of OA, but its role in the progression of OA has not been well established. Both biomechanical and metabolic factors are likely to link obesity and OA. Biomechanical overload on joints can accelerate cartilage degeneration, while metabolic dysfunction caused by obesity may have a more severe influence on OA than body weight [2]. Recent studies showed adipose tissue could increase cartilage degeneration through a secretion of pro-inflammatory mediators and synergistic regulation with other inflammatory cytokines [3].

The fat mass and obesity-associated (*FTO*) gene encodes a 2-oxoglutarate-dependent nucleic acid demethylase. Its structure has been reported, but the exact function of this gene remains unknown [4, 5]. Genetic studies have showed variants in *FTO* are associated with an increased body mass index (BMI) which is an obesity-related measurement in several studies [6–12]. A genome-wide association study (GWAS) named arcOGEN study had found that a *FTO* single-nucleotide polymorphism (SNP) rs8044769 was in the strongest association with female OA close to genome-wide significance (*P* = 6.85 × 10^− 8^). This significance was degenerative after BMI adjustment, hinting that this *FTO* polymorphism may exert its influence on OA through obesity [13, 14]. The association was replicated in a study on North American Caucasians [15]. A study on Chinese Han population denied this association, but the subject number of the Chinese study was a little bit small [16]. Until now, the association between FTO and OA remains debatable.

Therefore, we evaluated the potential association of the *FTO* polymorphism (rs8044769) with OA in the Chinese Han population with more cases and controls. We also evaluated the association of this polymorphism with BMI because of the known effect of FTO on BMI.

## Methods

### Subjects

All patients were recruited from the department of Sports Medicine and Adult Reconstructive Surgery, Drum Tower Hospital, School of Medicine, Nanjing University and were diagnosed as having knee OA according to the previous study [17]. All the radio-graphics were estimated by the Kellgren/Lawrence grading system [18]. Other knee diseases like rheumatoid arthritis, post-traumatic or post-septic arthritis, and developmental dysplasia of the knee were excluded. We set the youngest age for the controls which is the same with the cases. The control subjects were consecutively enrolled at the Physical Examination Center, Drum Tower Hospital Affiliated to the Medical school of Nanjing University. They had no sign or symptom of arthritis or joint diseases. All the study populations were non-institutionalized Han Chinese living in or around Nanjing. This study was approved by the ethics review committee of the Nanjing University and conducted according to the Declaration of Helsinki principles. All participants were informed with letter of consent.

### BMI measure

Height and weight were measured (without shoes and in light clothes) by medical technicians. BMI was calculated by taking a person’s weight and dividing it by their height squared. Subjects were classified as underweight (BMI < 18.5 kg/m^2^), normal (18.5 kg/m^2^ ≤ BMI < 25 kg/m^2^), overweight (25 kg/m^2^ ≤ BMI < 30 kg/m^2^), and obese (BMI ≥ 30 kg/m^2^) set by the criteria of World Health Organization [19].

### Genotyping

DNA was extracted from peripheral blood leukocytes using the NucleoSpin Blood QuickPure Kit (MACHEREY-NAGEL GmbH & Co. KG, Düren, German). The SNP was genotyped using the Taqman assay in an ABI 7500 system (Applied Biosystems, Foster City, CA, USA). Genotyping was done by laboratory personnel blinded to case status. Three percent of samples were duplicated, and the concordance rate was 100%.

### Statistical analysis

Characteristics of the OA and the control groups were evaluated by using chi-square and Mann-Whitney test. The Hardy–Weinberg equilibrium (HWE) of genotypes in OA and control groups was tested by using chi-square test. The differences of genotype and allele distributions were evaluated by using Chi-square test. The combined effect for the risk allele was evaluated by Mantel-Haenszel test. BMI among different genotypes of *FTO* polymorphism were compared using Kruskal-Wallis test. The Potential associations between BMI and OA were evaluated by using unconditional logistic regression modeling. All *P* value reported as statistical significance was set at < 0.05. Odds ratio (OR) was calculated with 95% confidence interval (95% CI). Calculations were performed using SPSS 12.5S (SPSS, Chicago, USA) or Stata 8.2 (Stata, College Station, TX, USA).

## Results

The basic characteristics of the study population were showed in Table 1. On average, OA cases were older and had greater BMI than controls. Females were prevalent in the OA group. The mean age was 62.51 ± 11.43 years and mean BMI was 25.76 ± 3.69 kg/m2 in cases compared to 54.07 ± 11.60 years and 24.91 ± 3.04 kg/m2 in controls, respectively. Genotype and allele frequencies for FTO polymorphism were not significantly different between OA cases and controls (Table 2). We combined our data with the data from the previous Chinese study and found no association between the risk allele with OA (Table 2) [16]. We next examined potential association of rs8044769 with OA when stratified by gender and BMI. No significant difference was observed in any comparisons between OA cases and controls (Table 3).

**Table 1:** Characteristics of the osteoarthritis and control study population

Characteristic	Cases	Controls
Sample size	890	844
Males (%)	226 (25.28)	681 (80.69)
BMI (mean ± SD)	25.76 ± 3.69	24.91 ± 3.04*
Underweight (%)	8 (0.89)	12 (1.42)
Normal weight (%)	400 (44.94)	422 (50)
Overweight (%)	376 (42.24)	368 (43.60)
Obese (%)	106 (11.91)	42 (4.98)
Age (mean ± SD)	62.51 ± 11.43	54.07 ± 11.60*

*BMI* body mass index (kg/m^2^), *OA* osteoarthritis, *K–L score* Kellgren/Lawrence score

underweight, BMI < 18.5; normal, 18.5 ≤ BMI < 25; overweight, 25 ≤ BMI < 30; obese, BMI ≥ 30

*P < 0.001 for Mann-Whitney test as applicable between cases/controls

**Table 2:** Genotype and allele frequencies of *FTO* polymorphism (rs8044769) in Han Chinese population

Subjects	*N*	Allele		*P* value	P_het_	OR (95% CI)	Genotype			*P* value
		C (%)	T (%)				CC (%)	CT (%)	TT (%)	
Cases	890	1096 (61.57)	684 (38.43)	0.436		0.947 (0.825–1.086)	333 (37.41)	430 (48.32)	127 (14.27)	0.438
Controls	844	1061 (62.85)	627 (37.14)				339 (40.16)	383 (45.38)	122 (14.46)	
Combined with another Chinese group										
Cases	890 + 1093	1096 + 233 (61.36)	684 + 153 (38.64)	0.478*	0.745**	0.958* (0.849–1.079)				
Controls	844 + 436	1061 + 528 (62.07)	627 + 344 (37.93)							

*P value and odds ratio were calculated by Mantel-Haenszel test, **P for heterogeneity

**Table 3:** Association of *FTO* polymorphism (rs8044769) with osteoarthritis when stratified by gender and BMI

Groups compared	CC vs. other combined			TT vs. other combined			C allele vs. T allele			All genotype
	*P* value	OR	95% CI	*P* value	OR	95% CI	*P* value	OR	95% CI	*P* value
Female cases (*n* = 665) vs. controls (*n* = 163)	0.802	0.956	0.673–1.36	0.402	0.819	0.513–1.31	0.801	1.03	0.805–1.32	0.604
Male cases (*n* = 225) vs. controls (*n* = 681)	0.160	0.799	0.584–1.09	0.666	1.098	0.718–1.68	0.222	0.878	0.702–1.09	0.373
Normal weight cases (*n* = 400) vs. controls (*n* = 422)	0.241	0.845	0.639–1.12	0.831	1.04	0.706–1.54	0.345	0.908	0.744–1.11	0.494
Overweight cases (*n* = 376) vs. controls (*n* = 368)	0.448	0.892	0.663–1.20	0.742	0.934	0.621–1.40	0.712	0.961	0.780–1.19	0.623
Obese cases (*n* = 106) vs. controls (*n* = 42)	0.782	1.11	0.532–2.31	0.616	1.32	0.450–3.85	0.954	0.985	0.583–1.66	0.790

Associations between rs8044769 and BMI were also evaluated. Genotype and allele frequencies for FTO polymorphism were not significantly different between overweight and obese cases and controls (Table 4). No significant associations were observed between overweight and obese cases and controls in females, males, OA cases and OA controls, respectively. We also compared BMI among different genotypes of rs8044769 in Han Chinese population. We found no significant difference of BMI among three genotypes of rs8044769. We then examined associations between rs8044769 and BMI in females, males, OA cases, and controls, respectively, and found no significant differences of BMI among three genotypes of rs8044769 (Table 5).

**Table 4:** Associations between FTO polymorphism and BMI when stratified by gender and OA status

Groups compared	CC vs. other combined			TT vs. other combined			C allele vs. T allele			All genotype
	*P* value	OR	95% CI	*P* value	OR	95% CI	*P* value	OR	95% CI	*P* value
Total overweight and obese cases (*n* = 892) vs. controls (*n* = 842)	0.867	0.984	0.811–1.19	0.901	1.012	0.778–1.33	0.856	0.987	0.861–1.13	0.983
Overweight and obese cases (*n* = 423) vs. controls (*n* = 405) in females	0.835	0.971	0.734–1.28	0.738	1.07	0.725–1.57	0.750	0.968	0.794–1.18	0.940
Overweight and obese cases (*n* = 469) vs. controls (*n* = 437) in males	0.973	0.995	0.762–1.30	0.882	0.972	0.670–1.41	0.959	1.00	0.831–1.22	0.985
Overweight and obese cases (*n* = 482) vs. controls (*n* = 408) in OA cases	0.818	1.03	0.786–1.36	0.966	1.01	0.692–1.47	0.888	1.01	0.837–1.23	0.967
Overweight and obese cases (*n* = 410) vs. controls (*n* = 434) in OA controls	0.707	0.948	0.720–1.25	0.886	1.03	0.701–1.51	0.731	0.966	0.793–1.18	0.931

**Table 5:** Comparison of anthropometric parameters among different genotypes of *FTO* polymorphism (rs8044769) in Han Chinese population

Items	Genotype (mean ± SD)			*P* value
	CC	CT	TT	
BMI (kg/m^2^)	25.35 ± 3.37	25.37 ± 3.47	25.27 ± 3.33	0.980
BMI in females (kg/m^2^)	25.37 ± 3.70	25.50 ± 3.88	25.46 ± 3.86	0.925
BMI in males (kg/m^2^)	25.34 ± 3.07	25.26 ± 3.06	25.10 ± 2.76	0.797
BMI in cases (kg/m^2^)	25.77 ± 3.62	25.78 ± 3.79	25.67 ± 3.53	0.946
BMI in controls (kg/m^2^)	24.94 ± 3.08	24.91 ± 3.02	24.86 ± 3.07	0.966

*P* values were analyzed using Kruskal-Wallis test

We last examined potential associations between BMI and OA (Table 6). Through the logistic regression modeling, we demonstrated a higher BMI (overweight and obese, BMI ≥ 25) was a risk factor for OA in the Chinese Han population (Additional files 1 and 2).

**Table 6:** Association between high body mass index and osteoarthritis

Groups	N		Unadjusted		Adjusted^a^	
	Cases	Controls	OR	95% CI	OR	95% CI
Normal	400	422				
Overweight (25 ≤ BMI < 30)	376	368				
Obese (BMI ≥ 30)	106	42	1.08**	1.05–1.11	1.06*	1.02–1.10

*P* values were analyzed using unconditional logistic regression

*P = 0.006; **P < 0.001

^a^Adjusted for age and gender

## Discussion

In our study population, the mean age of OA patients is significantly higher than that of controls, and the females are significantly more prevalent in OA cases. The mean BMI in OA patients was significantly higher than that in controls, and the logistic regression analysis also showed that BMI was associated with OA. Our data conformed to the previous reports that age, female gender, and overload were risk factors for OA [20, 21].

The association of this *FTO* polymorphism (rs8044769) with OA was not replicated in the Chinese Han population. The previous GWAS found rs8044769 in *FTO* was one of the signals close to genome-wide significance for OA in a European population. Our results did not show any positive associations between rs8044769 and OA, even after stratification by gender and BMI. We combined our data with the data from another Chinese study and found no associations between the risk allele of rs8044769 and OA either.

The association of the *FTO* polymorphism (rs8044769) with BMI was not replicated in the Chinese Han population either. The previous studies showed that FTO was associated with BMI [6–12], and two of them referred to rs8044769 as BMI-associated SNP [11, 12]. In the association analysis of rs8044769 with BMI, we found no association between rs8044769 and BMI. And the BMIs corresponding different genotypes of rs8044769 showed no significant difference.

There are several possible reasons for the negative association between rs8044769 and OA. The sample size of our study might be insufficient. But regarding the odds ratio of the risk allele in our data and the previous Chinese study, it is difficult to get a significant difference by enlarging sample size. The gender distribution was not matched in OA patients and controls. In our study the genotype distributions did not vary a lot between females and males, and no significant association was found after stratification by gender.

We think the ethnic difference might be an important reason for the negative result in both the association study between rs8044769 and OA and the association study between rs8044769 and BMI, as the allele frequency and genotype distribution rs8044769 were different between Chinese Han population and Caucasian population [13–16]. The negative association in our population cannot deny the association of rs8044769 with OA and BMI in other populations.

An extended study of the GWAS study revealed that the association between rs8044769 and OA was degenerated after stratification by BMI, and the association between rs8044769 and BMI was identified in this study [14]. There have been several reports for the association between FTO and BMI [6–12], and two of them referred to rs8044769 as BMI associated SNP [11, 12]. So, we suspected that the association between rs8044769 and BMI may contribute to the association between this SNP and OA in the GWAS study. However, in our study, rs8044769 was not associated with BMI, and it might be another reason for the negative association between rs8044769 and OA.

From our data we cannot get a conclusion that FTO is not associated with BMI in Chinese population as the allele and genotype differences of rs8044769 do not equal to changes of FTO function or FTO expression. Some other genetic variants in FTO might be associated with BMI or OA in Chinese population, and further studies are needed to evaluate the association of FTO with BMI and OA.

The associations among rs8044769, BMI, and OA drew attentions to some concern on current GWAS results. Shortcomings like the imprecise phenotype definitions, chosen criterion, and limited study populations in the GWAS may lead to some false-positive association [22–25]. Stratification by clinical parameters such as BMI can reduce the risk of false-positive result, but the cost-effectiveness is uneconomical in the primary study. Future studies in OA genetics are required using more accurate phenotype definition, improved case/control selecting standardization, and increased sample sizes as well as extending studies to different populations. In addition, functional characterization and studies of the risk loci can help us understand better of the OA genetics.

Our results were consistent with a previous study in Chinese population and had enlarged studied subjects. We also conducted a meta-analysis together with the previous study in Chinese population to offer a solid evidence that rs8044769 of FTO was not associated with OA risk or BMI in Chinese population. By comparing with the result of the extended study of the GWAS study, we proposed that stratification by clinical parameters was crucial to reduce false-positive result in OA association studies, especially in GWAS studies with large-scale subjects.

Some limitations should be noted in our study. First, the number of study subjects of our study was still limited, although it was much larger than that in the previous Chinese study [16]. The sample size had a statistical power of 0.27 to detect an effect size of 1.10 with an *α*-level of 0.05 for the association of the SNP rs8044769 with OA risk. Secondly, we did not find detailed allele or genotype data for rs8044769 in the Caucasian reports, so we made a meta-analysis of only Chinese data. Considering the ethnic difference, our data cannot be generalized as a global result. Further studies in Chinese and other populations are needed to evaluate the association of this SNP with OA and BMI.

## Conclusion

The present study showed that the *FTO* polymorphism (rs8044769) was not associated with OA susceptibility in the Chinese Han population. This polymorphism was not associated with BMI in the Chinese Han population either. Higher BMI including overweight and obese is a risk factor for OA in the Chinese Han population. Further studies are necessary to identify the association of FTO with OA in different populations and to proclaim better of the OA pathogenesis.

## Additional files

Additional file 1: **Table S1.** Genotype and allele frequencies of *FTO* polymorphism (rs8044769) for association analysis on OA in Han Chinese population when stratified by gender and BMI. (DOC 47 kb)

Additional file 2: **Table S2.** Genotype and allele frequencies of FTO polymorphism (rs8044769) for association analysis on BMI in Han Chinese population when stratified by gender and OA status. (DOC 44 kb)

## Abbreviations

95% CI: 95% confidence interval
BMI: Body mass index
*FTO*: Fat mass and obesity-associated
GWAS: Genome-wide association study
HWE: Hardy–Weinberg equilibrium
OA: Osteoarthritis
OR: Odds ratio
SNP: Single-nucleotide polymorphism
