# Supplementary material for: FTO variant is not associated with osteoarthritis in the Chinese Han population: replication study for a genome-wide association study identified risk loci
Source: J Orthop Surg Res. 2018 Apr 2;13:65. doi: 10.1186/s13018-018-0769-2 (PMC5879643; doi:10.1186/s13018-018-0769-2)
Supplement: Supplementary file 1 — Table S1. Genotype and allele frequencies of FTO polymorphism (rs8044769) for association analysis on OA in Han Chinese population when stratified by gender and BMI. (DOC 47 kb) [file 13018_2018_769_MOESM1_ESM.doc]

**Additional file 1 Table S1 Genotype and allele frequencies of *FTO* polymorphism (rs8044769) for association analysis on OA in Han Chinese population when stratified by gender and BMI**

| Subjects | N | Genotype | | |  | Allele |  |  | Hardy-Weinberg equilibrium |
| --- | --- | --- | --- | --- | --- | --- | --- | --- | --- |
|  |  | CC (%) | CT (%) | TT (%) |  | C (%) | T (%) |  | P-Value |
| Cases | 890 | 333  (37.41) | 430  (48.32) | 127  (14.27) |  | 1096 (61.57) | 684  (38.43) |  | 0.531 |
| Controls | 844 | 339  (40.16) | 383  (45.38) | 122  (14.46) |  | 1061 (62.85) | 627  (37.14) |  | 0.413 |
| Female Cases | 665 | 254  (38.20) | 318  (47.82) | 93  (13.98) |  | 826 (62.11) | 504  (37.89) |  | 0.681 |
| Female Controls | 163 | 64  (39.26) | 72  (44.17) | 27  (16.57) |  | 200 (61.35) | 126  (38.65) |  | 0.381 |
| Male Cases | 225 | 79  (35.11) | 112  (49.78) | 34  (15.11) |  | 270 (60.00) | 180  (40.00) |  | 0.579 |
| Male Controls | 681 | 275  (40.38) | 311  (45.67) | 95  (13.95) |  | 861 (63.22) | 501  (36.78) |  | 0.638 |
| Normal weight cases | 400 | 148  (37.00) | 194  (48.50) | 58  (14.50) |  | 490 (61.25) | 310  (38.75) |  | 0.664 |
| Normal weight Controls | 422 | 173  (40.10) | 190  (45.02) | 59  (13.98) |  | 536 (63.51) | 308  (36.49) |  | 0.556 |
| Overweight cases | 376 | 139  (36.97) | 184  (48.94) | 53  (14.09) |  | 462 (61.44) | 290  (38.56) |  | 0.525 |
| Overweight Controls | 368 | 146  (39.67) | 167  (45.38) | 55  (14.95) |  | 459 (62.36) | 277  (37.64) |  | 0.523 |
| Obese cases | 106 | 43  (40.57) | 47  (44.34) | 16  (15.09) |  | 133 (62.74) | 79  (37.26) |  | 0.595 |
| Obese Controls | 42 | 16  (38.10) | 21  (50.00) | 5  (11.90) |  | 53 (63.10) | 31  (36.90) |  | 0.633 |
