# Supplementary material for: FTO variant is not associated with osteoarthritis in the Chinese Han population: replication study for a genome-wide association study identified risk loci
Source: J Orthop Surg Res. 2018 Apr 2;13:65. doi: 10.1186/s13018-018-0769-2 (PMC5879643; doi:10.1186/s13018-018-0769-2)
Supplement: Supplementary file 2 — Table S2. Genotype and allele frequencies of FTO polymorphism (rs8044769) for association analysis on BMI in Han Chinese population when stratified by gender and OA status. (DOC 44 kb) [file 13018_2018_769_MOESM2_ESM.doc]

**Additional file 2 Table S2 Genotype and allele frequencies of FTO polymorphism (rs8044769) for association analysis on BMI in Han Chinese population when stratified by gender and OA status**

| Subjects | N | Genotype | | |  | Allele |  |  | Hardy-Weinberg equilibrium |
| --- | --- | --- | --- | --- | --- | --- | --- | --- | --- |
|  |  | CC (%) | CT (%) | TT (%) |  | C (%) | T (%) |  | P-Value |
| Total overweight & obese | 892 | 344 (38.57) | 419  (46.97) | 129  (14.46) |  | 1107 (62.05) | 677  (37.95) |  | 0.938 |
| Total normal weight controls | 842 | 328  (38.96) | 394  (46.79) | 120  (14.25) |  | 1050 (62.35) | 634  (37.65) |  | 0.923 |
| Overweight & obese in females | 423 | 161 (38.06) | 199  (47.05) | 63  (14.89) |  | 521 (61.58) | 325  (38.42) |  | 0.906 |
| Normal weight controls in females | 405 | 157  (38.77) | 191  (47.16) | 57  (14.07) |  | 505  (62.35) | 305  (37.65) |  | 0.928 |
| Overweight & obese in males | 469 | 183 (39.02) | 220  (46.91) | 66  (14.07) |  | 586 (62.47) | 352  (37.52) |  | 0.993 |
| Normal weight controls in males | 437 | 171  (39.13) | 203  (46.45) | 63  (14.41) |  | 545 (62.35) | 329  (37.65) |  | 0.826 |
| Overweight & obese in OA cases | 482 | 182 (37.76) | 231  (47.92) | 69  (14.31) |  | 595 (61.72) | 369  (38.28) |  | 0.754 |
| Normal weight controls in OA cases | 408 | 151  (37.01) | 199  (48.78) | 58  (14.22) |  | 501  (61.40) | 315  (38.40) |  | 0.559 |
| Overweight & obese in OA controls | 410 | 162 (39.51) | 188  (45.85) | 60  (14.63) |  | 512 (62.44) | 308  (37.56) |  | 0.650 |
| Normal weight controls in OA controls | 434 | 177  (40.78) | 195  (44.93) | 62  (14.29) |  | 549  (63.25) | 319  (36.75) |  | 0.485 |
